# Rezafungin for Salvage or Consolidation Therapy of Invasive Fungal Disease: Experience in Real‐World Clinical Practice

**DOI:** 10.1111/myc.70163

**Published:** 2026-02-20

**Authors:** Jorge Boán, Eduardo Aparicio‐Minguijón, Mario Fernández‐Ruiz, María Asunción Pérez‐Jacoiste Asín, Isabel Rodríguez‐Goncer, Francisco López‐Medrano, Rafael San‐Juan, José Tiago Silva, Ana Pérez‐Ayala, Jose Manuel Caro‐Teller, José María Aguado

**Affiliations:** ^1^ Unit of Infectious Diseases Hospital Universitario 12 de Octubre, Instituto de Investigación Hospital 12 de Octubre (imas12) Madrid Spain; ^2^ Centro de Investigación Biomédica en Red de Enfermedades Infecciosas (CIBERINFEC) Instituto de Salud Carlos III (ISCIII) Madrid Spain; ^3^ School of Medicine Universidad Complutense Madrid Spain; ^4^ Department of Microbiology Hospital Universitario 12 de Octubre, Instituto de Investigación Sanitaria Hospital 12 de Octubre (imas12) Madrid Spain; ^5^ Department of Pharmacy Hospital Universitario 12 de Octubre, Instituto de Investigación Sanitaria Hospital 12 de Octubre (imas12) Madrid Spain

**Keywords:** candidemia, invasive candidiasis, real‐world experience, rezafungin

## Abstract

**Background:**

Echinocandins are the first‐line treatment for invasive candidiasis. Rezafungin is a novel echinocandin with improved pharmacokinetic properties that allow for once‐weekly intravenous administration, offering potential advantages in complex clinical settings.

**Objectives:**

We aimed to describe our real‐world experience with rezafungin for the treatment of invasive fungal disease (IFD).

**Patients:**

A retrospective analysis of consecutive patients treated with rezafungin for proven IFD at our center between September 2022 and December 2025 was conducted.

**Results:**

We included 13 patients (mean age: 59.4 ± 17.8 years). Main predisposing conditions included immunosuppression (53.8% [solid organ transplantation in 30.8%]), solid cancer (30.8%) and recent surgical intervention (38.5%). Most frequent *Candida* species were *C. albicans* (38.5%), *C. parapsilosis* (30.8%) and *Nakaseomyces glabratus* (15.4%). One patient had necrotizing gingival infection due to *Trichoderma longibrachiatum.* Main types of IFD included intravascular *Candida* infection (38.5%) and surgical‐site and intra‐abdominal candidiasis (23.1% each). Reported reasons for initiating rezafungin were the presence of fluconazole‐resistant isolates (53.8%), facilitating outpatient antifungal therapy (30.8%) and avoiding anticipated drug–drug interactions with triazoles (15.4%). No patient received rezafungin as a first‐line treatment and most (92.3%) had been previously exposed to other echinocandins. Rezafungin was typically initiated upon clearance of follow‐up blood cultures (80.0%). Median number of weekly doses was 4.0 (interquartile range: 3.0–5.5). Clinical cure (complete or partial response) by day +30 was achieved in all evaluable patients (100.0% [12/12]), with no treatment‐emergent adverse events.

**Conclusions:**

Rezafungin was effective and safe as salvage or consolidation therapy in patients with IFD, including deep‐seated candidiasis.

**Abbreviations**
ECMM: European Confederation of Medical Mycology
EHR: electronic health record
EORTC‐MSG: European Organisation for Research and Treatment of Cancer and the Mycoses Study Group
ESCMID: European Society of Clinical Microbiology and Infectious Diseases
HSCT: haematopoietic stem‐cell transplantation
IDSA: Infectious Diseases Society of America
IMD: invasive mould disease
IQR: interquartile range
IV: intravenous
MIC: minimum inhibitory concentration
OPAT: outpatient parenteral antimicrobial therapy
PK: pharmacokinetics
RCT: randomised clinical trial
SD: standard deviation
SOT: solid organ transplantation
TEAE: treatment‐emergent adverse event
TRAE: treatment‐related adverse event

## 1 Introduction

Despite advances in diagnostic methods and therapeutic approaches, invasive candidiasis and its most common form, candidemia, remain a relevant cause of morbidity and death [1]. Beyond the historically high all‐cause mortality rates (above 30% across recent multicentre studies [2, 3]), a worldwide progressive shift towards non‐*albicans Candida* species with reduced susceptibility to azoles has been observed, such as *Nakaseomyces glabratus* ( *C. glabrata* ), *C. parapsilosis* , or *C. auris* (now *Candidozyma auris*) [4, 5]. In addition, the management of invasive candidiasis entails a significant economic burden, with a relevant component attributable to hospitalisation costs [6].

Echinocandins exhibit potent in vitro activity against a broad range of *Candida* species, with an excellent safety profile and few drug–drug interactions [7]. This class of antifungals has been recommended as the first‐line therapy for invasive candidiasis and candidemia by the European Society of Clinical Microbiology and Infectious Diseases (ESCMID) and the European Confederation of Medical Mycology (ECMM) [8, 9] and Infectious Diseases Society of America (IDSA) guidelines [10] published during the past decade. Some invasive mould diseases (IMDs) may also benefit from echinocandin‐containing regimens [11, 12]. Limitations of first‐generation echinocandins include the requirement for daily intravenous (IV) administration and moderate penetration into the peritoneal fluid in cases of intra‐abdominal candidiasis [7, 13].

Rezafungin is a next‐generation, long‐acting echinocandin with improved pharmacokinetics (PK) allowing for once‐weekly IV dosing [14], which would facilitate its outpatient administration and contribute to reducing hospital stay and associated costs [15]. Rezafungin shows in vitro activity against *Candida* spp. (including azole‐resistant strains), *Aspergillus* spp. and *Pneumocystis jirovecii* [16, 17]. In phase 2 and 3 randomised clinical trials (RCTs), rezafungin was shown to be non‐inferior to caspofungin in adult patients with candidemia or other forms of invasive candidiasis [18, 19, 20]. Additional theoretical advantages include the enhanced microbiological efficacy over extended periods of time [21] and the lack of dose adjustments in special populations [22, 23].

Data on the effectiveness and safety of rezafungin outside the setting of pivotal RCTs [18, 19, 20] and case reports [24, 25, 26, 27, 28] are scarce, with only some published experiences in candidemia, invasive candidiasis, and selected IMD treated within the early access program [29, 30, 31]. Therefore, the present study aims to describe the real‐world prescription practices and outcomes in consecutive patients treated with rezafungin at our centre during the past 3 years.

## 2 Patients and Methods

### 2.1 Study Population and Setting

In the present retrospective cohort study, we included all adult patients (≥ 18 years) diagnosed with invasive fungal disease (either invasive candidiasis or IMD) who received at least one IV dose of rezafungin between September 2022 and December 2025 at the University Hospital ‘12 de Octubre’ (Madrid, Spain). Eligible patients were identified through the prescription orders provided by the Department of Pharmacy. Next, patients’ electronic health records (EHRs) were retrospectively reviewed. Clinical information was collected by means of a standardised case report form that included patient demographics, comorbidities, type of invasive fungal disease, diagnostic approaches, fungal species identified and results of antifungal susceptibility testing, as well as the reason for initiating rezafungin treatment, previous exposure to antifungals, therapeutic response (as defined below), patient outcomes (attributable and all‐cause mortality), tacrolimus trough levels before the initiation of treatment and at weekly intervals while on rezafungin (when applicable) and occurrence of treatment‐emergent (TEAEs) and treatment‐related adverse events (TRAEs). Patients were followed up for 180 days from the initiation of treatment or until death or the end of study (31 December 2025), whichever occurred earlier.

The study protocol was approved by the institutional Clinical Ethics Committee and no informed consent was required due to its retrospective observational nature. The research was performed in accordance with the ethical standards outlined by the Declaration of Helsinki. All personal data were processed in an anonymized way according to applicable national laws and regulations.

### 2.2 Study Definitions and Procedures

Rezafungin was given as a single IV loading dose of 400 mg, followed by 200 mg on day +7 and once weekly thereafter. The overall response to rezafungin treatment was assessed based on clinical, radiological and mycological criteria and was further categorised as complete response, partial response, stable disease, progression, or death according to the European Organisation for Research and Treatment of Cancer and the Mycoses Study Group (EORTC‐MSG) consensus [32]. The documentation of complete or partial response by day + 30 was considered as clinical cure, whereas the remaining outcomes (stable disease, progression or death regardless of attribution) were considered as failure. These outcomes were retrospectively adjudicated by two investigators (J.B. and E.A.M.) and reviewed by two senior researchers (M.F.R. and J.M.A.). Day 0 was defined as the day on which the first dose of rezafungin was administered.

The certainty of diagnosis was based on the last update of the EORTC‐MSG criteria [33] and only proven cases of invasive fungal disease were considered. Immunosuppression was defined by the presence of solid organ transplantation (SOT), haematopoietic stem‐cell transplantation (HSCT), active haematological malignancy, solid organ cancer receiving cytostatic chemotherapy, neutropenia (absolute neutrophil count < 0.50 × 10^3^ cells/μL), corticosteroid therapy (prednisone ≥ 20 mg daily or equivalent dose for more than 1 week, or at a lower dose for more than 3 months), or the use of any other immunosuppressive agent within the previous 3 months.

In vitro antifungal susceptibility testing was performed for *Candida* by Sensititre YeastOne and for *Trichoderma* by E‐test performed at the Hospital and confirmation by the EUCAST E.Def 9.4 reference method performed at the National Centre of Microbiology [34]. Sensitivity to rezafungin was extrapolated from sensitivities to other echinocandins for which antifungal testing could be performed since the determination of the minimum inhibitory concentration for rezafungin is not yet available.

### 2.3 Statistical Analysis

Due to the low number of patients included, only descriptive analysis was conducted. Quantitative results were expressed as the mean ± standard deviation (SD) or the median and interquartile range (IQR). Qualitative data was expressed with absolute and relative frequencies. Repeated measurements for tacrolimus levels were compared with the Wilcoxon signed‐rank test. Analyses were performed using SPSS software version 22.0 (IBM Corp., Armonk, NY).

## 3 Results

### 3.1 Patient Characteristics

We included 13 patients who received 13 courses of rezafungin during the study period. Most of them (92.3% [12/13]) had invasive candidiasis, whereas the remaining patient had IMD due to *Trichoderma longibrachiatum*. This latter case has been reported in detail [35]. There was male predominance (61.5% [8/13]), with a mean age of 59.4 ± 17.8 years. Seven patients (53.8%) had some form of immunosuppression (mainly due to SOT or allogeneic HSCT). Surgical intervention within the previous month was present in 5 (38.5%) patients. *Candida albicans* was the most frequently isolated pathogen (38.5% [5/13]), followed by *C. parapsilosis* (30.8% [4/13]) and *Nakaseomyces glabratus* (15.4% [2/13]) (Table 1).

**TABLE 1:** Demographics, clinical characteristics and fungal isolates (*n* = 13).

Variables	
Age, years (mean ± SD)	59.4 ± 17.8
Male gender (*n* [%])	8 (61.5)
Major comorbidities and predisposing conditions (*n* [%])	
Surgical intervention in the previous month	5 (38.5)
Chronic liver disease	3 (23.1)
Diabetes mellitus	3 (23.1)
Coronary heart disease	3 (23.1)
Valvular heart disease	2 (15.4)
Immunosuppression^a^	7 (53.8)
Solid organ transplantation^b^	4 (30.8)
Solid organ cancer^c^	4 (30.8)
Haematological malignancy^d^	1 (7.7)
Fungal species isolated and AST (*n* [%])	
*Candida albicans*	5 (38.5)
Echinocandin‐resistant	0/5
Fluconazole‐resistant	1/5
*Candida parapsilosis*	4 (30.8)
Echinocandin‐resistant	0/4
Fluconazole‐resistant	3/4
*Nakaseomyces glabratus*	2 (15.4)
Echinocandin‐resistant	0/2
Triazole‐resistant	0/2
*Candida tropicalis*	1 (7.7)
Echinocandin‐resistant	0/1
Fluconazole‐resistant	1/1
*Trichoderma longibrachiatum*	1 (7.7)
Echinocandin‐resistant	0/1
Triazole‐resistant	1/1
Amphotericin B‐resistant	0/1
Type of invasive fungal disease (*n* [%])	
Intravascular infection	5 (38.7)
*Candida* infective endocarditis^e^	2/5
*Candida* vascular graft infection	1/5
Catheter‐related candidemia	1/5
Candidemia of abdominal source	1/5
Time to clearance of candidemia in follow‐up blood cultures, days (median [IQR])	7 (3–27)
*Candida* organ/space surgical‐site infection	3 (23.1)
Intra‐abdominal invasive candidiasis	3 (23.1)
Infected necrosis after acute pancreatitis	1/3
Liver abscess after radiofrequency ablation of HCC	1/3
*Candida* peritonitis due to gastric perforation	1/3
*Candida* esophagitis	1 (7.7)
Necrotizing gingival IMD	1 (7.7)

Abbreviations: AST, antifungal susceptibility testing; HCC, hepatocellular carcinoma; IQR, interquartile range; IMD, invasive mould disease; SD, standard deviation.

^a^ Includes solid organ transplantation (*n* = 4), cytostatic chemotherapy (*n* = 2), and allogeneic haematopoietic stem‐cell transplantation (*n* = 1).

^b^ Includes kidney (*n* = 3) and simultaneous kidney‐pancreas transplantation (*n* = 1).

^c^ Includes HCC (*n* = 2), pancreatic adenocarcinoma with malignant biliary obstruction (*n* = 1) and metastatic ovarian adenocarcinoma (*n* = 1).

^d^ Includes chronic myelomonocytic leukaemia that underwent allogeneic haematopoietic stem‐cell transplantation (*n* = 1).

^e^ Includes endocarditis after transcatheter aortic valve replacement (*n* = 1) and aortic prosthetic valve endocarditis (*n* = 1).

Regarding the type of invasive fungal disease, intravascular infections accounted for 38.7% (5/13) of cases: prosthetic infective endocarditis (*n* = 2), abdominal aortic endograft infection (*n* = 1), catheter‐related candidemia (*n* = 1) and candidemia of abdominal origin (*n* = 1). Surgical‐site infection accounted for 23.1% (3/13) of cases, whereas intra‐abdominal candidiasis was present in 23.1% (3/13). One further patient had refractory *Candida* esophagitis and the remaining one was diagnosed with necrotizing gingival infection due to *T. longibrachiatum* (Table 1).

### 3.2 Prior Antifungal Therapy

All patients had been exposed to systemic antifungal agents before the initiation of rezafungin. Echinocandins were the most frequently used (92.3% [12/13]; median: 28.5 days [IQR: 11.8–45.5]), followed by fluconazole (46.2% [6/13]; median: 24.5 days [IQR: 14.5–47.3]) and liposomal amphotericin B (30.8% [4/13]; median: 26.5 days [IQR: 15.3–43.8]). Individual trajectories of previous antifungal exposure are detailed in Figure S1 of Supplementary Results.

Fluconazole resistance was present at variable rates across *Candida* isolates (20.0% [1/5] for *C. albicans* [1/5], 75.0% [3/4] for *C. parapsilosis and* 100.0% [1/1] for *C. tropicalis* ), whereas no echinocandin resistance was identified. The *T. longibrachiatum* isolate was resistant to all triazoles with the exception of voriconazole.

### 3.3 Details of Rezafungin Treatment

Rezafungin was administered once weekly for a median of 4 weeks (IQR: 3.0–5.5). Two patients were still on therapy at the time of writing. The main reasons recorded in the EHRs for initiating rezafungin were as targeted therapy in the presence of fluconazole‐resistant isolates (53.8% [7/13]), to facilitate outpatient antifungal therapy to reduce the length of hospitalisation (30.8% [4/13]) and to avoid the anticipated risk of drug–drug interactions with triazoles (15.4% [2/13]) (Table 2). No patient received rezafungin as a first‐line treatment. In the subgroup of patients with intravascular infection, rezafungin was typically initiated upon clearance of candidemia in follow‐up blood cultures (80.0% [4/5]), which occurred at a median of 7 days (IQR: 3–27). Rezafungin was used in monotherapy in all cases of invasive candidiasis and concurrently with voriconazole for 4 weeks in the case of *Trichoderma* infection.

**TABLE 2:** Characteristics of courses of rezafungin treatment (*n* = 13).

Variables	
Previous exposure to antifungal agents	
Echinocandins (*n* [%])	12 (92.3)
Duration, days (median [IQR])	28.5 (12.8–45.5)
Fluconazole (*n* [%])	6 (46.1)
Duration, days (median [IQR])	24.5 (14.5–47.3)
Liposomal amphotericin B (*n* [%])	4 (30.8)
Duration, days (median [IQR])	26.5 (15.3–43.8)
Reasons for rezafungin treatment (*n* [%])	
Involvement of fluconazole‐resistant isolates	7 (53.8)
Outpatient antifungal therapy	4 (30.8)
Anticipated risk of drug–drug interactions	2 (15.4)
Number of weekly IV doses administered (median [IQR])	4.0 (3.0–5.5)
Type of antifungal regimen (*n* [%])	
Monotherapy	12 (92.3)
Combination therapy	1 (7.7)
Response in evaluable patients (*n* [%])	
Clinical cure by day +30	13/13 (100.0)
Clinical cure by day +90^a^	11/11 (100.0)
Clinical cure by day +180^b^	8/10 (80.0)
All‐cause mortality (*n* [%])	2 (14.4)
Attributable mortality (*n* [%])	0 (0.0)

Abbreviations: IQR, interquartile range: IV, intravenous.

^a^ Clinical cure by day +90 was not evaluable in two patients as they had not reached this follow‐up point at the end of study (31 December 2025).

^b^ Clinical cure by day +180 was not evaluable in three patients as they had not reached this follow‐up point at the end of study (31 December 2025). Two patients with non‐attributable death qualified as failure.

### 3.4 Treatment Outcomes

Clinical cure (i.e., complete or partial response) by day +30 was achieved in all patients (100.0% [13/13]). After a median follow‐up of 152.0 days (IQR: 78.5–190.0), all‐cause mortality was 14.4% (2/13), with no deaths attributable to invasive fungal disease. In detail, one patient died on day +96 due to progression of underlying leukaemia, whereas the second patient died on day +141 as a result of multiple postoperative complications following intestinal perforation. In both cases the planned course of rezafungin therapy had been completed. Therefore, clinical cure by day +180 days among evaluable patients (i.e., those with enough follow‐up time at the end of study) was 80.0% (8/10), as the two fatal cases qualified as failure per EORTC‐MSG criteria [32].

Rezafungin was well tolerated in all 13 patients and there were no TEAEs or TRAEs reported. In the subgroup of SOT and HSCT recipients (*n* = 5), there were no significant differences in median tacrolimus trough levels from baseline (8.6 ng/mL [IQR: 5.0–10.9]) to day +7 (7.2 ng/mL [IQR: 4.7–12.5]; *p*‐value = 0.465) and day +14 (8.9 ng/mL [IQR: 5.3–11.7]; *p*‐value = 1.000).

## 4 Discussion

Rezafungin, a new‐generation echinocandin, represents an advancement in the treatment of invasive candidiasis and candidemia, particularly in light of the rising antifungal resistance among non‐*albicans* species [4]. Growing evidence indicates that rezafungin has substantial in vitro activity against difficult‐to‐treat pathogens that usually exhibit low susceptibility or intrinsic resistance to azoles, such as *C. auris* , *N. glabratus* , or *C. krusei* (now *Pichia kudriavzevii*) [36, 37].

The use of rezafungin for invasive candidiasis is supported by a robust clinical development program that has been the basis for regulatory approval. The pivotal phase 3 ReSTORE trial established that once‐weekly rezafungin was non‐inferior to standard once‐daily caspofungin for the primary endpoints of all‐cause mortality by day +30 and global cure by day +14, with a comparable safety profile [19]. This confirmed the findings of the previous phase 2 RCT, which validated the once‐weekly dosing regimen [18]. The pharmacological rationale for this extended dosing interval is underpinned by its long elimination half‐life (around 130 h), metabolic and chemical stability (with no evidence of biotransformation in liver microsomes or hepatocytes), high protein binding and prolonged post‐antifungal effect [38].

In our experience with 13 patients with invasive fungal disease, many of them with some form of immunosuppression and prolonged previous exposure to antifungals, the use of rezafungin was associated with a rate of clinical cure of 100.0% by day +30. This result favourably compares with the rates of all‐cause mortality (18.7% by day +30) and mycological eradication (73.4% by day +5) reported for the rezafungin group of the ReSTORE trial [20]. It should be noted, however, that none of our patients received rezafungin as primary treatment, but rather as consolidation or salvage therapy. This reflects the preferential role for this agent in current clinical practice. Similarly to other reports [29, 39], rezafungin was primarily used to treat azole‐resistant isolates—a situation in which echinocandins remain the treatment of choice—and to reduce the length of hospitalisation through outpatient antifungal therapy. This latter point underscores the practical advantage provided by its distinctive PK profile [38].

Rezafungin was used with the primary aim of avoiding drug–drug interactions with triazoles in two patients. Akin to first‐generation echinocandins, this agent does not undergo significant hepatic metabolism and is not a substrate, inhibitor or inducer of any of the cytochrome P450 isoenzymes or major drug transporter proteins. The lack of expected drug interactions is particularly relevant in SOT and HSCT recipients, which accounted for one third of our cohort. No significant differences in the exposure parameters (C_max_ and area under the curve) of oral tacrolimus—a CYP3A4 and P‐glycoprotein substrate—were observed with the coadministration of rezafungin as compared with administration alone in healthy subjects [40]. Beyond anecdotal case reports under compassionate use [41], real‐world experiences with rezafungin for SOT recipients are essentially lacking. Of note, we found no differences in tacrolimus levels during the course of rezafungin treatment, which would support its safety in the transplant population.

The present series diverges from pivotal RCTs in relevant aspects that may inform the clinical use of rezafungin. The prevalence of *C. parapsilosis* isolates was high (30.8% of cases). Although no echinocandin resistance was detected, this pattern differs from the STRIVE and ReSTORE trials, in which *C. albicans* was predominant [18, 19, 20]. We included a significant proportion of patients with deep‐seated invasive candidiasis, which are rarely represented in registrational trials. Although intravascular infection does not constitute a well‐established indication for rezafungin, we obtained successful outcomes in three patients with prosthetic endocarditis and aortic endograft infection. This experience aligns with case reports of difficult‐to‐treat infections associated with biofilm formation, such as vertebral osteomyelitis [26], prosthetic joint infection [31] or prosthetic valve endocarditis [27, 28]. It has been shown that echinocandins exert good activity against *Candida* biofilms [42, 43, 44]. In a recent study, rezafungin exerted potent in vitro activity against non‐*parapsilosis Candida* planktonic cells, although significant fold‐change increases were observed for biofilm‐associated MICs [45].

A singular off‐label use of rezafungin was the treatment of an allogeneic HSCT recipient with necrotizing gingivitis due to *T. longibrachiatum*. Long considered non‐pathogenic in humans, *Trichoderma* spp. have been recognised as a rare cause of IMD in heavily immunocompromised haematological patients [46]. As recently described in detail by our group [35], the isolate had low MIC values to echinocandins, providing a microbiological rationale for rezafungin treatment in combination with voriconazole. There is limited data available on the anti‐mould activity of rezafungin and its role in the treatment of IMD remains to be defined. While in vitro preclinical studies suggest that rezafungin has activity against *Aspergillus* spp. comparable to other echinocandins [38, 47], we are aware of only one registered ongoing RCT for the treatment of chronic pulmonary aspergillosis in patients with limited therapeutic options (NCT06794554).

The duration of rezafungin treatment in our cohort (median of 4.0 weekly doses) was sensibly longer than the regimens tested in RCTs [19, 20] and closer to the real‐life multicentre experience recently published within the FungiScope registry (median of 9 weeks) [29]. This is concordant with the inclusion of deep‐seated infections requiring prolonged antifungal therapy [8, 9, 10]. In line with pivotal trials and clinical experience [27, 31, 39], no relevant safety or tolerability issues were observed, even in patients that received more than 4 weeks of therapy.

Our study has limitations inherent to its observational and retrospective design, including the small sample size and the lack of a control group. Furthermore, the selection of patients to receive rezafungin might have been biased towards complex cases with favourable outcomes expected. Therefore, the high rate of clinical cure observed should be taken with caution. Nevertheless, ours is the largest real‐life experience from a single center reported to date and includes a relevant representation of patient populations (SOT recipients) and indications (intravascular *Candida* infections) that are often excluded from RCTs.

In conclusion, the present experience demonstrates that rezafungin is both effective and safe for the treatment of invasive candidiasis, in accordance with the evidence derived from RCTs, while also exploring its role in other complex clinical scenarios. Its once‐weekly dosing regimen positions it as a valuable tool for consolidation therapy, facilitating hospital discharge and outpatient management. By detailing patient outcomes in a real‐life setting, our findings contribute to the evolving understanding of the utility and safety of this novel antifungal.

## Author Contributions

**Jorge Boán:** conceptualization, investigation, methodology, data curation, formal analysis, writing – original draft, writing – review and editing.

## Funding

This research was supported by an unrestricted grant from Mundipharma Pharmaceuticals Spain. J.B. and E.A‐.M. hold ‘Río Hortega’ research training contracts (CM25/00250 and CM25/00243) and I.R‐.G. holds a post‐doctoral research contract (JR24/00034), all from the Instituto de Salud Carlos III (ISCIII), Spanish Ministry of Science, Innovation and Universities. These funding sources had no role in study design, data collection and analysis, interpretation of data, manuscript preparation or decision to submit. The corresponding author had full access to all the data in the study and had final responsibility for the decision to submit it for publication.

## Disclosure

J.B.P. has received support for attending meetings from Shionogi, Takeda, Angelini and Pfizer. E.A‐.M has received support for attending meetings from Pfizer, Gilead and MSD. M.F.R. has received research funding from Pfizer Spain and honoraria for educational activities from Pfizer, MSD and Gilead Sciences. I.R‐.G. has received honoraria for educational activities from Pfizer and support for attending meetings from Pfizer. F.L‐.M. has received honoraria for educational activities from Mundipharma. R.S‐.J. has received honoraria for educational activities from Pfizer, Almirall and Alfasigma and support for attending meetings from Amgen, Novartis, Lilly, Pfizer and Takeda. J.M.C‐.T. has received honoraria for educational activities from Pfizer, Almirall and Alfasigma and support for attending meetings from Amgen, Novartis, Lilly, Pfizer and Takeda. The remaining authors have no conflicts of interest to disclose.

## Supporting information


**Figure S1:**Individual trajectories of previous antifungal exposure in 13 patients treated with rezafungin.

## Data Availability

The data that support the findings of this study are available from the corresponding author upon reasonable request.
