# Supplementary material for: Rezafungin for Salvage or Consolidation Therapy of Invasive Fungal Disease: Experience in Real‐World Clinical Practice
Source: Mycoses. 2026 Feb 20;69(2):e70163. doi: 10.1111/myc.70163 (PMC12922572; doi:10.1111/myc.70163)
Supplement: Supplementary file 1 — Figure S1:Individual trajectories of previous antifungal exposure in 13 patients treated with rezafungin. [file MYC-69-e70163-s001.docx]

**Supporting Material**

*Supplementary Results*

-
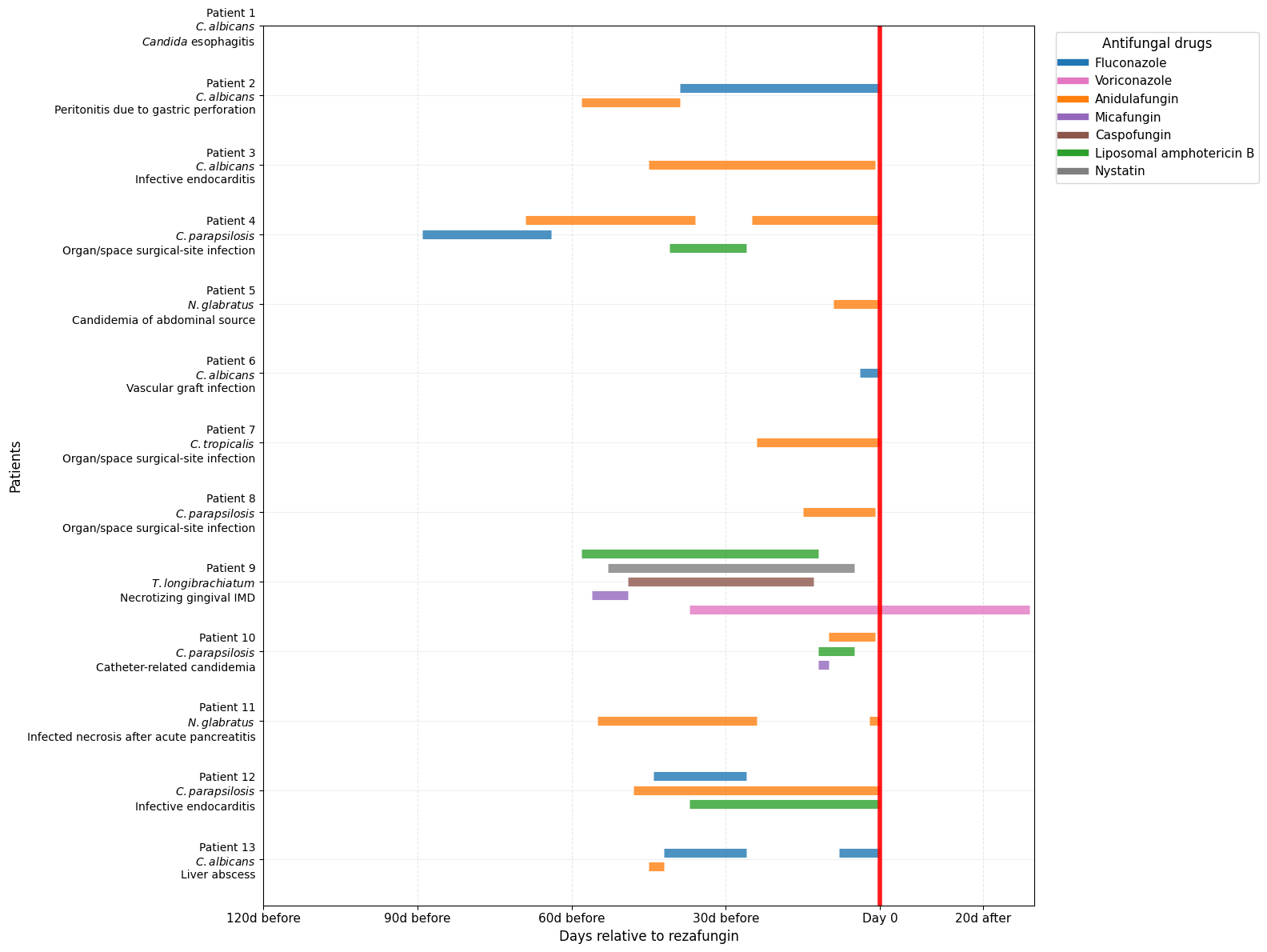
**Figure S1.** Individual trajectories of previous antifungal exposure in 13 patients treated with rezafungin.
